# The Details of Splint Application

**Published:** 1919-08-30

**Authors:** 


### PLASTER WORK.

### I. The Details of Splint Application.

In the modern, open-air, or conservative treatment of surgical tuberculosis, more especially in children, there is great scope for fixation of tuberculous joints by means of plaster of paris, and accordingly th technique of this medium has been much developed of late. When by rest in bed with extension apparatus muscular spasm has been overcome and faulty position, if present, corrected, and quiescence of the lesion assured, plaster of paris is undoubtedly an excellent form of splint. However, the details of its application are important. To begin with, ready-made and rolled bandages are unsuitable, if only for reasons of expense. Common book muslin, procurable at most haberdashers, should be used. For a hip it may be torn into six-inch strips. Plaster powder that has been baked for two or three hours before use is put into a tin box, and the loosely rolled strip of book muslin introduced and re-rolled the reverse way in the powder. By this means enough is incorporated in the bandage. The child, previously bathed and carefully dried, is clad in stockinette combinations, and lies on four supports, one for head and shoulders, one for each foot, and a very small one for the lower part of the sacrum. Begin round the lower part of the thigh in a simple spiral, but when nearing Poupart's ligament make over the middle of the thigh, and in the region of the great trochanter, a sort of reverse. That is, let the bandage return on itself a couple of inches, and then take it on in the original direction round the leg. These V-shaped "reverses" are built well up in the region of the groin, and then the bandage is taken, as in an ordinary spica, round the iliac spines. Before it is dry mould it with the thumbs round the iliac crests and down Poupart's ligament. If great strength is needed add a little Portland cement to the plaster powder, although this increases the weight somewhat. The plaster is to be applied in the early morning, and the child put to bed with hot-water bottles. Next day cut with scissors the combinations within an inch or so of the plaster above and below. Rub into the selvedge left some very thick plaster, of the consistency of cream, and turn it up on to the dry plaster. A smooth edge is thus secured, which will not cut or chafe. Remove the "dinner pad" which, of course, has been placed over the lower abdomen and cut a small "window" there, using a knife such as leather-workers sell. The last stage is polishing the plaster with some more "cream." A very little should be done at a time. The polished surface keeps much cleaner than a rough one would.

For a spinal jacket the child should stand on tiptoe, steadying itself with each hand on a support, and have the head lightly strung up by a band passing under occiput and under chin to a frame above. Combinations and "dinner pad" as before. The meal previous should be light, and the bowels should be cleared by enema. Begin bandaging below, and make it thick round the pubis. The "reverses" should come over the middle of the body; they act now not only as strengtheners, but as safety valves, relaxing a little with the movements of respiration and forming an additional security against over-tightness. When the bandage reaches to the axillæ continue it over both shoulders like a double "Sam Browne" belt. Before it is dry mould it to the clavicles, the scapulæ, and the iliac crests, thus ensuring a good fit. Finish just as before, also with an abdominal window.

The complications after the splint is finished may be roughly divided into three. It may not set well or may crack; tightness may be complained of; plaster sores may develop under it. The first contingency, which is very unlikely after the precautions enumerated, may yet happen in spite of the best management. Obviously re-application is indicated. The second may be met by a little "nicking." In an ankle bandage this should be in front of one malleolus and behind the other, corresponding to the anatomy of the veins of that region. Abdominal distension may be relieved by an enema. Sores under the plaster are either superficial (chafes) or deep (active tuberculous disease). The best way to detect them is by the nose; it is a good thing to smell plaster splints now and again. If the sore is of any extent a "window" should be cut over it and dressing applied in the usual way. In a concluding article we give the technique of the light and comfortable, but very efficient, celluloid splints, which commonly follow upon plaster fixation just as that does upon rest in bed with extension.

